# The Effect of High Molecular Weight Hyaluronic Acid and Latanoprost Eyedrops on Tear Functions and Ocular Surface Status in C57/BL6 Mice

**DOI:** 10.3390/jcm12020544

**Published:** 2023-01-09

**Authors:** Murat Dogru, Takashi Kojima, Kazunari Higa, Ayako Igarashi, Haruka Kudo, Wolfgang G. K. Müller-Lierheim, Kazuo Tsubota, Kazuno Negishi

**Affiliations:** 1 Department of Ophthalmology, Keio University School of Medicine, Tokyo 160-8582, Japan; 2 Department of Ophthalmology, Tsurumi University, Tsurumi, Yokohama 230-8501, Japan; 3 Department of Ophthalmology, Tokyo Dental College Ichikawa General Hospital, Ichikawa 272-8513, Japan; 4 i.com medical GmbH, 81241 Munich, Germany; 5 Tsubota Laboratory, Inc., Shinjuku-ku, Tokyo 160-0016, Japan

**Keywords:** dry eye, anti-glaucoma eye drops, hyaluronic acid eye drops, wild type mice

## Abstract

Anti-glaucoma eye drop treatment often induces ocular surface problems, including dry eyes, and may be associated with poor medication compliance. This study aimed to investigate the effects of a novel high molecular weight hyaluronic acid and Latanoprost eye drop on intraocular pressure, as well as the tear function and ocular surface alterations in wild type mice, comparing the results with the mice receiving commercially available Latanoprost eye drops and mice receiving no treatment. The mice were divided into three groups: Group I, control group (no treatment group); Group II, commercial Latanoprost eye drop (LP); and Group III, Comfort Shield (CS) + Latanoprost (LP) eye drop (CS + LP). The CS + LP eye drop group had an IOP lowering effect comparable to the commercial LP eye drop group. The mice receiving LP eye drops had significantly worse corneal staining scores, lesser goblet cell density(GCD), higher numbers of CD45+ staining cells, significantly higher tear film concentrations of IL-6 and IL1-b, and a significantly lower expression of corneal ZO-1 mRNA compared with the mice receiving CS + LP 7 days after eye drop instillations (*p* < 0.05). In conclusion, the new CS + LP formulation appeared to induce less inflammation, less corneal vital staining, and a better barrier status with an IOP lowering effect comparable to the commercial LP eye drops.

## 1. Introduction

Glaucoma affects more than 60 million people globally, with 8.4 million people being bilaterally blind [1]. Long-term use of topical anti-glaucoma eye drops and the control of intraocular pressure is still the mainstay of treatment to protect the optic nerve from further damage. Treatment entails the use of topical eye drops that enhance the aqueous outflow, reduce the aqueous production, or both. Latanoprost is a prostaglandin analogue that increases scleral permeability to the aqueous humor and aqueous outflow. It is preferred as the first line of treatment for ocular hypertension including open angle glaucoma. The literature indicates many reports on the preservative induced toxic ocular surface effects of anti-glaucoma eye drops [2,3,4,5,6,7,8,9,10,11,12]. Glaucoma-related ocular surface disease can present with superficial punctate keratitis, tear-film instability, allergy, pseudopemphigoid, and dry eye disease, which have been linked to poor medication compliance [13]. Therefore, finding efficient ways of managing the ocular surface disease in such patients remains a priority in this field. The current approach for managing such ocular surface disorders involves the use of non-preserved anti-glaucoma eye drops, as well as the addition of non-preserved artificial tears or lubricants, including hyaluronic acid eye drops or mucin secretagogues such as diquafosol sodium or rebamipide eye drops, into the treatment regimen. Other modalities of management include the use of topical anti-inflammatory agents such as cyclosporine and lifitegrast, autologous serum eye drops, or the insertion of punctal plugs [13,14]. The use of new and novel drugs, agents, or delivery systems is very much needed to alleviate the ocular surface problems associated with antiglaucoma drops.

Previously, we investigated and reported the favorable anti-inflammatory and neurotrophic effects of very high molecular weight hyaluronic acid (vHMWHA) in ocular surface diseases associated with dry eyes [15], which gave us the idea to conjugate vHMWHA with the first line treatment agent in glaucoma, namely, Latanaprost. Our hypothesis was to test whether such a new formulation could help with the alleviation of the ocular surface disease associated with glaucoma treatment. In this study, we investigated the effect of a novel vHMWHA and Latanoprost eye drop on intraocular pressure, as well as the tear function and ocular surface alterations in wild type mice, comparing the results with mice receiving commercially available Latanoprost eye drops and mice receiving no treatment.

## 2. Materials and Methods

### 2.1. Animals

Fifteen eyes of fifteen 6~8-week-old C57BL/6 background mice were investigated in Phase 1, and twenty-four eyes of twenty-four 6~8 week-old C57BL/6 background mice were investigated in Phase 2 of the study. The mice were obtained from Clea, Osaka, Japan. No abnormality was found in the anterior segment when examined with the slit lamp microscope at the time of arrival to the TDC animal facility. The mice were housed in the same standard environment conditions throughout the study. All of the procedures were performed in accordance with the Association for Research in the Vision and Ophthalmology Statement for the Use of Animals in Ophthalmic and Vision Research. The eyes of the mice were divided into three groups in Phase 1 as follows:

Group 1 (Control): Control group without the application of eye drops.

Group 2 (LP): Commercial Latanaprost eye drops once a day for 1 week. The formulation contained boric acid, trometamol, polyoxyethylene castor oil, EDTA, and a pH modifier, with an LP concentration of 50μg/mL and an osmolarity of 276 mOsm.

Group 3 (CS + LP): Very high molecular weight hyaluronic acid + Latanaprost eye drops once a day for 1 week. The formulation contained vHMWHA, sodium chloride (NaCl), disodium hydrogen phosphate (Na_2_HPO_4_), sodium dihydrogenphosphate (NaH_2_PO_4_), and purified water, with an LP concentration of 14 μg/mL and an osmolarity of 255 mOsm.

In Phase 2, there were eight mice in each group. All of the mice also had body weight measurements taken throughout the study. Eye drop instillations were carried out at the same time every day between 16:00–17:00 p.m. The IOP measurement experiments (Phase 1) and ocular surface examinations (Phase 2) were carried out as two separate studies in two phases because repetitive IOP measurements would have affected the tear film and ocular surface status.

### 2.2. Examination Procedures

Phase 1 study: The IOP assessment study.

Phase 1 involved the measurement of IOPs at baseline (day 0) and on day 7. IOP was measured using an Icare ic100: Rebound Tonometer (ICT). The Icare ic100 tonometer is a portable rebound tonometer that relies on the induction or impact principle. Mainly, the device consists of six parts, including a forehead support, forehead adjusting wheel, probe, display, navigation button, and measure button. The Icare tonometry stainless-steel probe is 50 mm long and includes two coaxial magnet systems with a diameter of 1.4–1.0 mm. In order to obtain an accurate measurement, the tonometer was positioned at 4 mm horizontally from the central cornea. The voltage formed in the magnetic system by the corneal contact with the movement of the probe was detected by the sensor and transformed into a digital signal. The probe tip was covered with plastic to minimize the risk of corneal damage. Six consecutive measurements were taken and the mean of these six measurements was automatically calculated. Eye drop instillations were done at the same time for each mouse on each of the days. All IOP measurements were performed at the same time on days 0 and 7 by the same examiner.

Phase 2 study: Tear function and ocular surface assessments.

**Figure 1:**
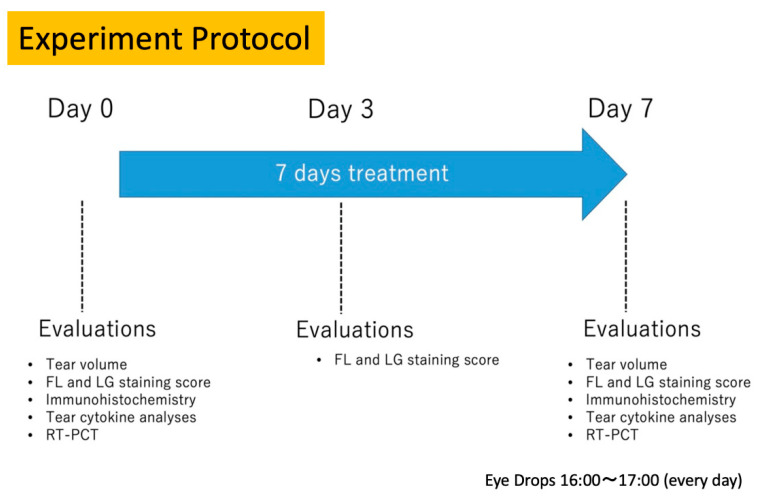
Diagram showing the experimental protocol for the Phase 2 ocular surface safety study.

The scheme of the Phase 2 protocol, for the tear function and ocular surface tests performed on days 0, 3, and 7, are shown in Figure 1. The ocular surface evaluations on day 3 were not the focus of the study, but were done to confirm if an ocular surface epitheliopathy/OS disorder was induced by the glaucoma eye drops. Our focus was to compare the tear functions and OS examinations before and after treatment.

### 2.3. Tear Collection and Tear Cytokine Analyses

Phenol red-impregnated cotton threads (Zone-Quick, Showa Yakuhin Kako Co., Ltd., Tokyo, Japan) were immersed into the tear meniscus in the lateral canthus of the mice eyes for 60 s and then placed in 1.5 mL Eppendorf tubes and stored at −80 degrees until the tear cytokine analyses. The veritas cytokine assay was performed according to the user manual instructions. Briefly, the samples underwent assay dilution following the standard dilution set up and the set up for blank and control wells. After the biochip was fixed to the 384-well plate base frame, 40 microliters of wash buffer 1 was added to the wells with a 16-channel pipette. Following centrifuge, the plate was left for incubation at room temperature for 15 min. After taping the plate over a paper towel, the plate was centrifuged in the reverse direction briefly to get rid of excess solution. Five microliters of the sample solutions were applied to the biochips and centrifuged. The plate was sealed and left for incubation at room temperature for 3 h. Following tapping on a paper towel, 40 microliters of wash buffer 1 was added to each well with a multichannel pipette. This process was repeated three times. Ten microliters of the detection antibody solution was added to each well and the plate was centrifuged. The biochip was sealed and incubated at room temperature for 1 h. Following tapping, 40 microliters of wash buffer 1 was added to each well and removed. This process was again repeated three times followed by a reverse centrifuge to remove the excessive solution. Ten microliters of Streptavidin-PE (SA-PE) solution was added to each well and the plate was centrifuged. The biochip was again sealed and incubated at room temperature for 30 min. Following tapping, 40 microliters of wash buffer 1 was added to each well and removed. This process was repeated five times. Finally, 40 microliters of wash buffer 2 was added to each well. Following tapping and centrifuge, the plate was covered with an aluminum foil to avoid light exposure and was placed in a desiccator for 30 min for complete the dry-up. The plate was then placed in a LunarisTM Reader(AYOXXA Biosystems, Koln, Germany) for determining the cytokine levels. The IL-1b and IL-6 levels were measured and the concentrations were expressed in pg/mL.

### 2.4. Tear Volume Measurements

Phenol red-impregnated cotton threads (Zone-Quick, Showa Yakuhin Kako Co., Ltd., Tokyo, Japan) were used to measure the aqueous tear production without anesthesia. Briefly, cotton threads were immersed into the tear meniscus in the lateral canthus of the mice eyes for 60 s and the length of the wet thread was measured in millimeters.

### 2.5. Corneal Vital Stainings

A total of 2 μL of 0.5% sodium fluorescein and 1% LG was instilled into the conjunctival sac. Corneal epithelial damage was assessed after 2 min of dye instillation. Fluorescein staining tests were conducted using a hand-held slit lamp biomicroscope using cobalt blue light (Kowa, Tokyo, Japan). For scoring the Fluorescein and Lissamine green stainings, the mice corneas were divided into three equal zones, namely upper, middle, and lower zones. Each zone had a staining score ranging between 0 and 3 points, with the minimum and maximum total staining scores ranging between 0 and 9 points. The presence of scarce staining in one zone was scored as one point, whereas punctate staining covering the entire zone was scored as three points.

### 2.6. Conjunctival Specimen and Eyeball Collections

The mice were anesthetized intraperitoneally and were sacrificed using 50 mg/mL of pentobarbital (Kyoritsu Seiyaku Co., Tokyo, Japan) after 1 week of eye drop instillations. The whole globes were rapidly removed after being sacrificed. For histopathologic assessment, the globes were fixed in the Tissue-Tek OCT compound (Sakura Finetec, Tokyo, Japan) and frozen with liquid nitrogen, and then stored in a −80 °C freezer.

### 2.7. Conjunctival PAS Staining and Goblet Cell Density Calculations

For PAS staining, 5-micron thick conjunctival sections were processed according to conventional histological techniques. Briefly, after fixation with acetone at 4 °C for 10 min, the slides were washed with distilled water and immersed in 0.5% periodic acid for 5 min, rinsed in three changes of distilled water for 5 min, and immersed in Schiff solution for 10 min. The slides were then exposed to 0.5% sodium bisulfite solution for 3 min twice. The slides were rinsed with distilled water again for 5 min and underwent hematoxylin staining for 1 min. The slides were rinsed with distilled water for 15 min and then coverslipped. We counted the goblet cells in three randomly selected nonoverlapping areas in each section at 200× magnification using a light microscope and recorded the numbers. The average goblet cell density was calculated for each slide and was recorded as the number of cells/high power field.

### 2.8. Immunofluorescense Staining (IF) for Conjunctival muc5AC and CD45 and Inflammatory Cell Calculations

The anti-muc5AC (ab3649) mouse monoclonal antibody (Abcam, Cambridge, UK) diluted with donkey blocking serum at 1:100 was used. The CD45 (ab10558) rabbit polyclonal antibody diluted with donkey serum at 1:150 was used for the conjunctival inflammatory cell staining. Tissue sections were cool dried and fixed with acetone for 10 min at room temperature, and washed with PBS for 5 min three times. The samples were marked with a PAP pen. The slides were then incubated with normal donkey serum (Merck, St.Louis, MO, USA) for 30 min at room temperature to block the nonspecific background staining. The tissues were then treated with the primary rabbit polyclonal antibody at 4 °C and incubated overnight. For the negative controls, the primary antibody was replaced with the rabbit IgG isotype control at the same concentration of the primary antibody (ab37415) (ABCAM, Cambridge, UK). The sections were then washed with PBS for 5 min three times. The sections were then treated and incubated with anti-Rabbit IgG-FITC Donkey antibody (Jackson’s Lab, West Grove, Penna, USA) for 30 min at room temperature under aluminum foil cover to avoid light exposure. The sections were then washed in PBS for 10 min three times in a prepared DAPI chromogen solution (1:1000 dilution) (Thermo Fisher Scientific, Waltham, MA, USA), washed with PBS for 2–3 min three times, dehydrated, and mounted. Sections were then evaluated and imaged using an Axioplan2 imaging microscope (Carl Zeiss, Jena, Germany).

### 2.9. Immunofluorescence Staining (IF) for Corneal ZO-1

The anti-ZO-1 mouse monoclonal antibody (ThermoFisher, Waltham, MA, USA) diluted with donkey blocking serum at 1:100 was used. The tissue sections were cool dried and fixed with acetone for 10 min at room temperature, and washed with PBS for 5 min three times. The samples were marked with a PAP pen. The slides were then incubated with normal donkey serum (Melck, Darmstadt, Germany) for 30 min at room temperature to block the nonspecific background staining. The tissues were then treated with the primary mouse monoclonal antibody at 4 °C and incubated overnight. For the negative controls, the primary antibody was replaced with the mouse isotype control at the same concentration of the primary antibody (33-9100) (Thermo Fisher, Waltham, MA, USA). The sections were then washed with PBS for 5 min three times. The sections were then treated and incubated with the secondary antibody, the anti-mouse IgG-FITC Donkey (Jackson’s Lab, West Grove, Penna, USA), for 30 min at room temperature under an aluminum foil cover to avoid light exposure. The sections were then washed in PBS for 10 min three times, developed in a prepared DAPI chromogen solution (1:1000 dilution) (Thermo Fisher Scientific, Waltham, MA, USA), washed with PBS for 2–3 min three times, dehydrated, and mounted. The sections were then evaluated and imaged using an Axioplan2 imaging microscope (Carl Zeiss, Jena, Germany).

### 2.10. Real Time RT-PCR for Corneal ZO-1 mRNA and Conjunctival muc5AC mRNA Expressions

The total RNA was isolated from the corneas and conjunctivas using an RNA extraction reagent (ISOGENII; Nippon Gene, Tokyo, Japan), according to the manufacturer’s instructions. Briefly, the samples were placed into a 1.5 mL tube and homogenized with 500 microliters of Isogen II solution. Then, 200 microliters of RNase free water was added and the samples were shaken vigorously for 15 s. The plate was centrifuged at 13,000 rpm for 15 min. Then, 500 microliters of the supernatant was collected and transferred to 1.5 mL Eppendorf tubes. Then, 2.5 microliters of 0.5% p-Bromoanisole solution was added and mixed for 15 s and left at room temperature for 5 min, followed by a centrifugation of 13,000 rpm for 10 min. The supernatants were removed and 500 microliters of 75% ethanol was added onto the pellets. Further centrifugation was done at 8000 rpm for 3 min. The addition and centrigation of alcohol was repeated again. The supernatants were totally removed and the pellets were dried for 5 min. The pellets were lysed with 30 microliters of RNase free water, which allowed for total RNA isolation. The RNA was used for reverse transcription, and then cDNA synthesis was performed. A SYBR green-based quantitative real-time PCR was performed using the Platinum SYBR Green qPCR SuperMix-UDG (Invitrogen, Waltham, MA, USA). The expression levels of the mRNA were evaluated using the ΔΔCt method and normalized to the level of glyceraldehyde-3-phosphate dehydrogenase (GAPDH). Each PCR amplification was performed using a specific primer set. The primers used were ZO-1 (eurofins:1668636-1), MUC5AC (invitrogen: 86366522: s1017), and GAPDH (invitrogen:85935148:R8306). The primer sequences were as follows:

GAPDH, 5′-TGACGTGCCGCCTGGAGAAA-3′(sense) and 5′-GACTTCCCGTAGAACCCGATGTGA-3′(antisense); ZO-1, GATAGTTTGGCAGCAAGAGATGGTA(sense), and AGGTCAGGGACGTTCAGTAAGGTAG (antisense).

Muc5ac: 5′-AAAGACACCAGTAGTCACTCAGCAA-3′(sense) 5′-ACCAAACTGTGACTGAAGGGTC-3′(antisense).

### 2.11. Statistical Analysis

Graphpad Instat-3 software (Graphad Instat Corp., San Diego, CA, USA) was used for the statistical analysis. One-way ANOVA test and t tests were employed for the statistical analyses. A *p* value less than 5% (*p* < 0.05) was considered to be statistically significant.

## 3. Results

### 3.1. IOP Measurements

**Figure 2:**
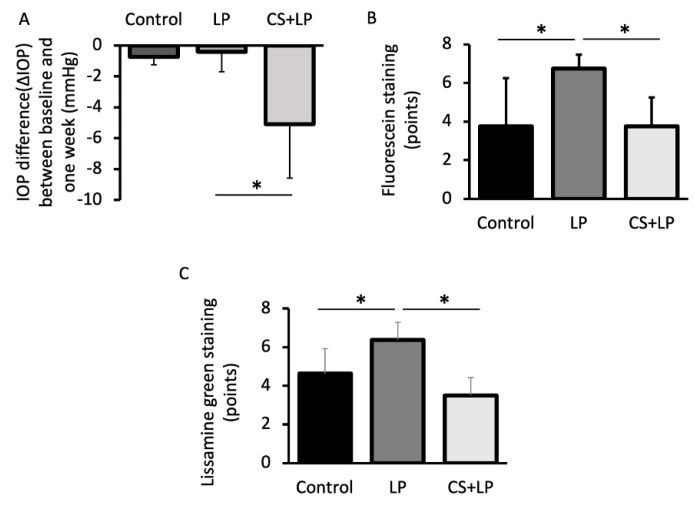
Comparison of the intraocular pressure difference and vital staining scores at one week. (**A**) Note the significantly greater ΔIOP in the Comfort Shield+ Latanoprost (CS + LP) compared with commercial Latanoprost (LP) group after one week of eye drop use (*p* = 0.02). (**B**,**C**): Note the significantly lower fluorescein and Lissamine green scores in Comfort Shield+ Latanoprost (CS + LP) group compared to Latanoprost (LP) group (*p* < 0.05) * represents *p* < 0.05.

All of the IOP measurements with ICT were performed by the same experienced clinician. The mean of IOP change in Groups 1, 2, and 3 were −0.75 ± 0.5 mmHg, −0.40 ± 1.3 mmHg, and −5.167 ± 3.54 mmHg, respectively. The mean of IOP change at 7 days was significantly greater in Group 3 compared with Group 2, as shown in Figure 2A.

### 3.2. Changes in Body Weight, Tear Quantity and Corneal Epithelial Vital Staining Scores

The mean body weight of the mice in each group did not show significant differences at 0 and 7 days and from baseline to 7 days in each group (*p* > 0.05). Likewise, the mean tear quantity of mice in each group did not show significant differences at 0 and 7 days and from baseline to 7 days in each group (*p* > 0.05). The mean FS scores in Groups 1, 2, and 3 at 3 days were 3.5 ± 1.5 points, 5.5 ± 1.25 points, and 4.6 ± 1.5 points, respectively. The mean LG scores in Groups 1, 2, and 3 at 3 days were 4.75 ± 2.75 points, 4.8 ± 2.2 points, and 5.25 ± 1.5 points, respectively. Figure 2 shows a comparison of the corneal fluorescein and Lissamine green vital staining scores. The mean FS scores in Groups 1, 2, and 3 at 7 days were 3.75 ± 2.5 points, 6.75 ± 0.75 points, and 3.75 ± 1.5 points, respectively. The mean LG scores in Groups 1, 2, and 3 at 7 days were 4.6 ± 1.3 points, 6.1 ± 1.0 points, and 3.5 ± 1.0 points, respectively. The mean FS and LG scores were significantly lower in the CS + LP group (Group 3) at 7 days compared with the LP group (Group 2) (Figure 2B,C).

### 3.3. Conjunctival PAS Staining, Goblet Cell Density Alterations, Conjunctival muc5 Stainings, and Real Time RT-PCR muc5 AC Expressions

**Figure 3:**
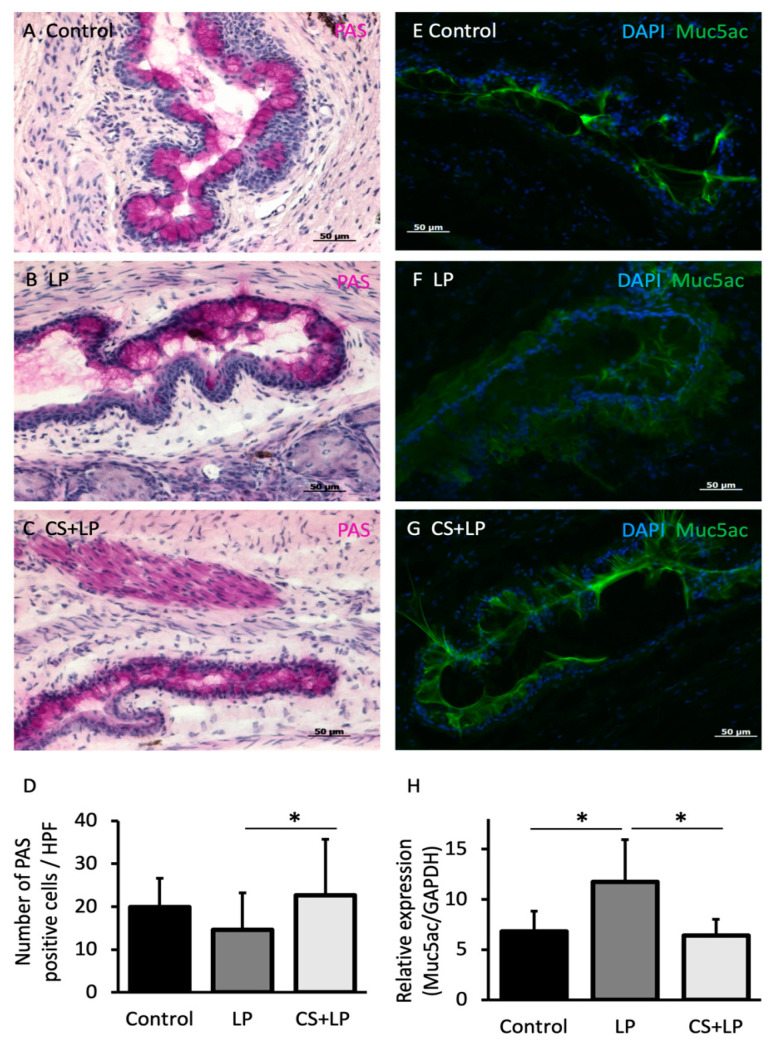
Conjunctival goblet cell periodic acid–Schiff (PAS) staining, muc5 immunohistochemistry staining, goblet cell density, and real time RT-PCR conjunctival muc5 mRNA expression comparisons. (**A**–**C**) Note the abundance of plump oval pink-purple goblet cells in the conjunctival epithelium in Group 1 (no treatment group) and Group 3, and the comparably scarce PAS positive goblet cells in Group 2 (Latanoprost group). (**D**) Note the significantly lower mean goblet cell density in the LP group compared with the CS + LP group (*p* < 0.01). (**E**–**G**) Note the intense staining for muc5 in the conjunctival epithelium in Group 1 (no treatment) and Group 3 (CS + LP) and the comparably less intense staining in Group 2 (LP). (**H**) Note the significantly increased muc5 mRNA expression in Group 2 (LP group) compared with Group 3 (CS + LP group) and Group 1 (no treatment group) (*p* = 0.011 and *p* = 0.0043, respectively). * represents *p* < 0.05.

Figure 3A–C shows the conjunctival PAS staining findings at 7 days for each group. Although conjunctival specimens in the mice belonging to Groups 1 and 3 showed abundant oval plump goblet cells, those in Group 2 showed a notable decrease in stained goblet cells. Figure 3D shows the comparison of GCD at 7 days. The mean number of goblet cells/HPF in Groups 1, 2, and 3 at 7 days were 20 ± 5 cells, 14 ± 6 cells, and 26 ± 9 cells, respectively. The mean GCD in Group 2 was significantly lower than those in Group 3 (*p* < 0.01). Figure 3E–G shows the conjunctival muc5 immunohistochemistry staining findings at 7 days for each group. The conjunctival specimens in Groups 1 and 3 showed abundant muc5 staining in the conjunctival epithelium with relatively less intense staining in Group 2. Figure 3H shows the muc5 mRNA expression comparisons after 7 days for each group. The muc5 mRNA expression level in Group 2 was significantly higher than that in Groups 1 and 3 (*p* = 0.0043 and *p* = 0.011, respectively).

### 3.4. Corneal ZO-1 and Conjunctival CD45 IHC Stainings and Real Time RT-PCR Alterations

**Figure 4:**
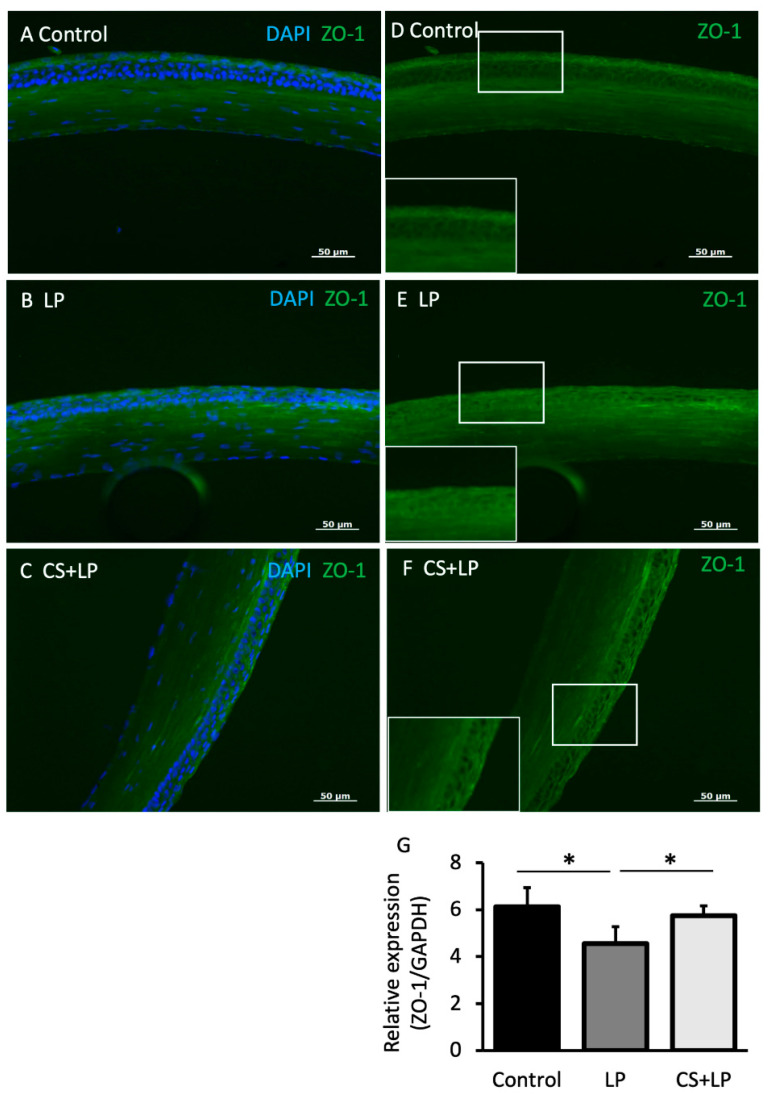
Corneal ZO-1 immunohistochemistry staining and real time RT-PCR corneal ZO-1 mRNA expression comparisons. (**A**–**C**) Note the marked staining for ZO-1 in the corneal epithelium in Group 1 (no treatment group) and Group 3 (CS + LP group), and the comparably lesser staining in Group 2 (LP group). (**D**–**F**) Lower left pictograms represent magnified images of the upper corneal square inserts. (**G**) Note the significantly decreased ZO-1 mRNA expression in the LP group compared with the CS + LP group and the no treatment group ( *p*= 0.011 and *p* = 0.003, respectively). * represents *p* < 0.05.

Figure 4A–F shows the corneal ZO-1 staining findings after 7 days for each group. While the corneal specimens in Groups 1 and 3 showed remarkable ZO-1 staining in the corneal epithelium, there is scanty ZO-1 staining in Group 2. Figure 4G shows the ZO1 mRNA expression comparisons after 7 days for each group. The ZO-1 mRNA expression level in Group 2 was significantly lower than that in Groups I and 3 (*p* = 0.011 and *p* = 0.003, respectively).

**Figure 5:**
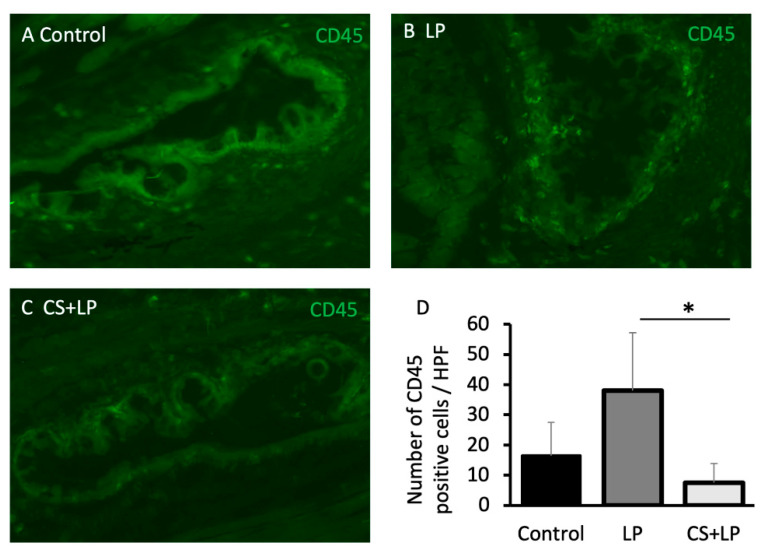
Conjunctival CD45 immunohistochemistry staining and CD45 positive conjunctival cell count comparisons. (**A**–**C**) Note the abundance of CD45 positive inflammatory cells in the conjunctival epithelium in Group 2 (LP group) compared with Group 3 (CS + LP group). (**D**) Note the significantly increased CD45 positive inflammatory cell counts in the LP group compared with the CS + LP group (*p* = 0.02). * represents *p* < 0.05.

Figure 5A–C shows the conjunctival CD45+ staining findings after 7 days for each group. While the conjunctival specimens in Groups 1 and 3 showed scanty CD45+ staining in a close vicinity to the conjunctival epithelium, there was abundant CD45+ staining in Group 2. Figure 5D shows the CD45 + staining cell count comparisons at 7 days in each group. The mean CD45+ cell counts/HPF in Groups 1, 2, and 3 after 7 days were 16 ± 9 cells, 40 ± 15 cells, and 7 ± 5 cells, respectively. The CD45+ cell count/HPF in the conjunctival specimens in Group 2 was significantly higher than that in Group 3 (*p* = 0.02).

### 3.5. Tear ELISA IL-6 and IL-1b Alterations

**Figure 6:**
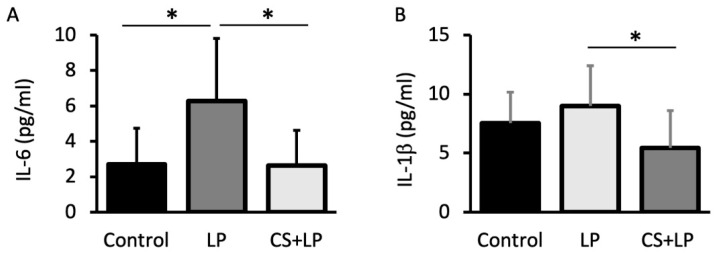
Tear IL-6 and IL-1b concentration comparisons after 7 days of eye drop use. (**A**) Note the significantly higher mean tear IL-6 concentration in Group 2 (LP group) compared with Group 1 (no treatment group) and Group 3 (CS + LP group) (*p* = 0.01 and *p* = 0.009, respectively). (**B**) Note the significantly higher mean tear IL-1b concentration in Group 2 (LP group) compared with Group 3 (CS + LP group) (*p* = 0.01). * represents *p* < 0.05.

Figure 6A,B shows the tear IL-6 and IL1-b concentrations after 7 days for each group. The mean tear IL-6 concentrations in Groups 1, 2, and 3 at 7 days were 2.5 ± 2.5 pg/mL, 6.0 ± 4.0 pg/mL, and 2.5 ± 2.5 pg/mL, respectively. The mean tear IL-6 concentration in Group 2 was significantly higher than that observed in Groups 1 and 3 (*p* = 0.01 and *p* = 0.009, respectively). The mean tear IL-1b concentrations in Groups 1, 2, and 3 at 7 days were 7.5 ± 2.5 pg/mL, 8.5 ± 4.0 pg/mL, and 5.5 ± 2.5 pg/mL, respectively. The mean tear IL-1b concentration in Group 2 was significantly higher than that observed in Group 3 (*p* = 0.01).

## 4. Discussion

Glaucoma treatment may induce ocular surface inflammation, resulting in a wide variety of ocular surface disorders. Glaucoma-therapy-related ocular surface disease (GROSD) has been defined as the “imbalance of the ocular surface homeostasis caused by the toxic effect of topical medications which leads to tear film instability, epithelial damage and inflammation” [16]. Previous studies have reported tear instability and reduced tear quantity in over 60% of glaucoma patients. The rate of punctate corneal erosions can be as high as 18–54% in patients receiving antiglaucoma eye drops [17,18,19,20,21,22]. On the severe end of the ocular surface disease (OSD) spectrum is pseudo-pemphigoid, which can be diagnosed in up to 28.3% of chronic eye drop users [23]. Increasing the number of antiglaucoma eye drops and the presence of preservatives have been reported to aggravate GROSD [6,24,25]. Switching to non-preserved anti-glaucoma eye drop regimens is known to improve the signs and symptoms of GROSD [26]. The approach to GROSD also entails prescribing non-preserved lubricants, anti-inflammatory agents such as cyclosporine and lifitegrast, autologous serum eye drops, the insertion of punctal plugs, or switching to surgical alternatives [13]. A previous study by us found better tear stability, lesser ocular surface epithelial damage, and higher goblet cell densities when 3% diquafosol sodium (mucin and water secretagogue) eye drops were started at the time of the commencement of anti-glaucoma eye drops [14]. In this ocular surface health assessment study, we aimed to study the short-term intraocular pressure and ocular surface alterations of a new formulation harboring a high molecular weight hyaluronic acid and Latanoprost in WT mice, comparing the results with mice receiving solitary non-preserved commercial LP and mice receiving no treatment. Our data from the Phase 1 study suggest that IOP change has a trend of a small decrease in the control and LP groups with a marked ΔIOP change in CS + LP group. A currently ongoing aqueous humor penetration study for the same formulation of LP and CS + LP and diluted commercial LP with PBS performed by our group suggest the impressive penetration of CS + LP into the anterior chamber, which has been sustained in the aqueous humor with comparable levels to LP. CS + LP appears to use a different receptor system for penetration than LP alone (unpublished data). While LP was expected to bring about a significant ΔIOP, only a mild reduction was observed in the current study. The limited number of eyes is a limitation of the current study and might explain the absence of significant IOP change between the LP and control groups. We believe that IOP measurements should be performed on a larger number of mice in the future.

Preserved and non-preserved LP eye drops are known to induce worsening of OSDI scores, tear film break up time, and ocular staining values in the first month of treatment, with no further worsening thereafter in clinical human trials [27]. In the Phase 2 study, mice receiving the CS + LP drops had a better ocular surface health status compared with the mice receiving commercial LP eye drop instillations at 7 days, as evidenced by the significantly lower corneal vital staining scores and higher conjunctival goblet cell densities. The corneal epithelial barrier appeared to be in a better condition in the CS + LP group based on the findings of a significantly higher ZO-1 mRNA expression (epithelial barrier protein), although data from future long term in vivo studies considering other barrier proteins are needed. The higher muc5 mRNA expression in the LP group can be attributed to a “hypothetical compensatory effect” by the reduced population of goblet cells producing higher amounts of muc5 to protect ailing ocular surface health due to a higher extent of corneal epithelial damage. This finding is contradictory to the general consensus for ocular surface mucins in glaucoma-drop-related ocular surface disease, where a reduction in mucin expressions is expected [28]. Compensatory mucin changes do occur, especially in atopic ocular surface disease associated with tear instability and corneal shield ulcers. In a previous study, we reported that there is a compensatory increase in MUC5AC expression just before and at the time of corneal ulceration in atopic keratoconjunctivitis (severe ocular surface disease), and the expression declines with the chronicity of inflammation [29]. The CS + LP group also had a significantly lower ocular surface-tear film unit inflammation, as evidenced by the significantly lower conjunctival CD45+ staining inflammatory cell counts and lower tear IL-6 and IL-1b levels. Explanations regarding why the mice in the CS + LP group had less inflammation, less corneal vital staining, and better barrier status may be due to a protective effect of the vHMWHA or a dilution effect by the HMWHA in the formulation. Further experimentation with the addition of a group such as “PBS + LP” would provide very useful information to further illustrate the protective effect of CS in eye drops.

Topical LP is known to promote the activation of P38-NFkB signaling and the production of inflammatory cytokines such as TNF-alpha, IL-1b, IFN-gamma, and IL-17, together with CD4* T cell infiltration in the conjunctiva. Topical LP also increases the expression of MMP-3 and MMP-9 and decreases the expression of ZO-1 and Occludin 1 in the corneal epithelium [28]. These effects have been suggested to translate into decreased tear production, loss of goblet cells, tear instability, and disruption of the corneal epithelial barrier in clinical practice, even with instillations of non-preserved LP eye drops [30].

Hyaluronic acid (HA) eye drops have been reported to have muco-protective and anti-inflammatory effects in in vivo animal and human studies [31]. HA reduces the OS friction from the blinks and influence aqueous retention. The higher the molecular weight of HA, the higher the viscosity attained at the ocular surface when the shear rate becomes small, i.e., vHMWHA eye drops should have a greater stabilizing effect on the tear film during eye opening [31]. The effect of HA on the immune response also depends on its MW, and vHMWHA is known for its remarkable anti-inflammatory effects. vHMWHA can bind strongly to membrane bound mucins and strengthen the ocular surface barrier against pathogens. In a previous study, Kojima et al. used an environmental desiccating stress induced dry eye mouse model. The Balb/c model mice were fitted into a narrow compartment in a temperature and humidity controllable room, placed 5 cm from an air fan, and exposed to continuous air flow (4 m/s) at a constant room temperature of 23 ± 2 °C and a constant humidity of 25 ± 5% for 3 days from 09:00 a.m. to 14:00 p.m. Kojima et al. showed that the use of vHMWHA eye drops was associated with better tear stability, vital staining scores, and better muc5 mRNA expression and lesser keratoconjunctival dendritic cells in confocal microscopy compared with the mice receiving low MWHA formulations [15]. HA induces its effects by binding to its cell membrane CD44 receptors and extracellular proteins called hyaladherins. HA-CD44 interactions control cellular processes such as inflammation, cell proliferation, migration, and wound healing [32,33,34]. The binding of HA to cellular hyaladherins depends on the molecular weight. The higher the MW of HA, the stronger the binding [30,31,32]. HMWHA also induces antioxidant and antiapoptotic effects, all of which might explain the less vital stainings, better goblet cell densities, higher ZO-1 mRNA expression, and less ocular surface and tear film inflammation in the CS + LP group compared with the solitary LP group. The better ocular surface status may also be because of a lower concentration of LP in the CS + LP formulation (approx. 3.5-fold) compared with the commercial LP eye drops. Still, the formulation decreased the intraocular pressure significantly within a week, and the change in IOP from baseline was also comparable to the commercial LP eye drops. Whether this effect was due to a better ocular surface penetration aided by the vHMWHA needs to be clarified by future invivo study designs investigating the LP concentration in the aqueous humor in a time-dependent fashion. Clinical trials such as the HYLAN-M study showed that vHMWHA provided better symptomatic relief and increased the corneal nerve fiber lengths in confocal microscopy in dry eye patients. The results of the HYLAN-M study suggested that vHMWHA could pass the cell barrier of the corneal epithelium and alter the extracellular matrix in the close vicinity of corneal nerves, improving the corneal trophism, or due to pharmacological downregulation of inflammation resulting in nerve recovery [35]. Although the scope of the current preliminary study was to study the ocular surface safety and health with the new formulation in WT mice, long term comparative instillation studies with various antiglaucoma eye drops and studies of IOP and ocular surface changes in glaucoma mouse models would provide very interesting information.

In summary, vHMWHA combined with LP is as safe as non-preserved LP eye drops as far as ocular surface health is concerned, and is associated with less corneal vital staining, better epithelial barrier, and less tear film and ocular surface inflammation.

## Data Availability

All data kept on lab notes at Ichikawa General Hospital, Tokyo Dental College, Ichikawa, Japan.
